# MiR-208b/miR-21 Promotes the Progression of Cardiac Fibrosis Through the Activation of the TGF-β1/Smad-3 Signaling Pathway: An *in vitro* and *in vivo* Study

**DOI:** 10.3389/fcvm.2022.924629

**Published:** 2022-07-05

**Authors:** Yifan Zhang, Bo Yuan, Yue Xu, Na Zhou, Ruiqi Zhang, Lan Lu, Zhanbin Feng

**Affiliations:** ^1^Department of Cardiology, Ninth Hospital of Xi’an, Xi’an, China; ^2^Stroke Centre and Department of Neurology, The First Affiliated Hospital of Xi’an Jiaotong University, Xi’an, China

**Keywords:** cardiac fibrosis (CF), miRNAs, signaling pathway, TGF-β1, Smad-3

## Abstract

**Background:**

Regulatory molecule microRNAs (miRNAs) have been implicated in myocardial fibrosis. However, the specific mechanism by which they lead to myocardial fibrosis remains unclear. This study aimed to explore the roles of miR-208b, miR-21 and transforming growth factor-β1 (TGF-β1)/Smad-3 signaling pathway components in cardiac fibrosis development.

**Materials and Methods:**

Thirty-six consecutive acute myocardial infarction (AMI) patients were included in this study. Plasma was collected on admission and at 24 h, 48 h and 6 d. The levels of plasma miR-208b, miR-21, TGF-β1, and Smad-3 were measured using reverse transcription-quantitative polymerase chain reaction (RT-qPCR), and cardiac calcium protein T (cTnT) and creatine kinase isoenzyme (CK-MB) were detected by electrochemiluminescence analysis. H9C2 cells were exposed to hypoxia and divided into 4 groups (hypoxia treatment for 6 h, 24 h, 48 h, and 72 h). These stimulated cells were then transfected with miRNA inhibitors and mimics for gene overexpression and inhibition. RT-qPCR was used to detect the expression of miR-208b, miR-21, TGF-β1, and Smad-3, and western blot analysis was used to detect TGF-β1 and Smad-3 protein expression.

**Results:**

The plasma analysis showed cTnT and CK-MB expression peaked at 24 h after symptom onset; miR-208b, miR-21, TGF-β1, and Smad-3 levels showed no peak and increased gradually with time. Cell experiments revealed that miR-208b and TGF-β1 were upregulated along with increased hypoxia exposure; miR-21 expression peaked at 24 h and 72 h, with the highest peak at 72 h, and Smad-3 expression peaked at 6 h and 72 h, with the highest peak at 72 h. miR-208b and miR-21 expressions were positively correlated with TGF-β/Smad-3 expression. TGF-β1/Smad-3 mRNA and protein levels were elevated in the miR-208b and miR-21 overexpression groups and reduced in the miR-208b and miR-21 inhibition groups.

**Conclusion:**

MiR-208b and miR-21 promote cardiac fibrosis progression through TGF-β1/Smad-3 signaling pathway activation.

## Introduction

Cardiovascular diseases are the leading cause of mortality worldwide. Myocardial cells have a limited ability to regenerate, and the death of ischemic myocardial cells will trigger a repair program; that is, damaged tissues will be replaced by fibrotic scars produced by myofibroblasts (1). Although initial cardiac fibrosis plays an important role in preventing ventricular wall rupture, excessive fibrosis in infarct zones and reactive fibrosis in non-infarct zones can lead to cardiac alterations in morphology, biomechanics and biochemistry, which will further damage cardiac function and ultimately lead to heart failure (2).

As regulatory molecules, microRNAs (miRNAs) regulate protein translation during transcription. These molecules are involved in almost all physiological and pathological processes, including cell proliferation, apoptosis and differentiation. Callis TE (3) indicated that miR-208b is encoded within the β-cardiac muscle myosin heavy chain gene (Myh7). This miRNA is cardiac-specific and participates in cardiac hypertrophy and fibrosis (4, 5). In addition, a study showed that miR-21 is highly expressed in fibrotic myocardium and that its expression increases with the severity of heart failure. Moreover, a miR-21 inhibitor blocked myofibroblast proliferation (6, 7). However, the precise functions of miR-21 and miR-208b in cardiac fibrosis are still unclear. TGF-β is a key mediator implicated in cardiac fibrosis (8), and activated TGF-β binds to TGF-β receptors, resulting in subsequent activation of its downstream target Smad-3, which promotes myofibroblast proliferation and migration and ultimately leads to cardiac fibrosis (9). To elucidate the molecular mechanism of cardiac fibrosis after acute myocardial infarction (AMI) regarding miR-208b/miR-21 via the TGF-β1/Smad-3 signaling pathway, we detected the expression levels of miR-208b/miR-21 and TGF-β1/Smad-3 in cells and blood samples from AMI patients and analyzed their relationships.

## Materials and Methods

### Study Population

AMI patients admitted to the cardiology department of the Ninth Hospital of Xi’an were enrolled from June 2019 to December 2020. The diagnosis of AMI was made according to the third universal definition of myocardial infarction from Thygesen et al. (10). The inclusion criteria were detection of a rise and/or fall of cardiac biomarker values [preferably cardiac troponin (cTn)] with at least one value above the 99th percentile upper reference limit (URL) and with at least one of the following: symptoms of ischemia, new or presumed new significant ST-segment–T wave (ST–T) changes or new left bundle branch block (LBBB), development of pathological Q waves in the ECG, imaging evidence of new loss of viable myocardium or new regional wall motion abnormality. Patients with coma, cardiac arrest, cardiogenic shock and severe systemic disease (such as severe infection, malignant tumor, severe hepatic and renal dysfunction) were excluded. Patients whose culprit vessel was unclamped were also excluded. All patients were diagnosed by surface ECG, cardiac ultrasound and blood biochemistry analysis. This study was approved by the Ethics Committee of the Ninth Hospital of Xi’an.

### Sample Collection

A total of 7 ml of blood from each patient was obtained immediately after admission and at 24 h, 48 h and 6 d after onset of the symptoms. The first 2 ml of blood was sent for biochemical analysis, and the remaining 5 ml of blood was centrifuged at 3500 × g for 30 min at 4°C. After 10 min, the serum was separated, packaged in 200 μl EP tubes and stored in a freezer at –80°C for future analysis.

### Cell Culture

H9C2 cells were ordered from Procell (China) and cultured in high glucose DMEM with 10% fetal bovine serum (FBS), 10 μM 5-aza-2’- deoxycytidine and antibiotics (Procell, China) at 37°C in 5% CO2. All the groups of cells were placed in a nitrogen incubator at 37°C, and 99.9% pure nitrogen was introduced to balance the oxygen in the incubator and were cultured for 6 h, 24 h, 48 h, and 72 h, respectively, and according to different hypoxic culture times, the cells were divided into 4 groups (hypoxia treatment for 6 h, 24 h, 48 h, and 72 h).

### Cell Transfection

Conventional hypoxic treatment was used for the control group, and miRNA overexpression and inhibition groups were established in all different hypoxic culture groups. MiRNA inhibitors and mimics were purchased from RiboBio (Guangdong, China). Twenty-four hours before treatment, the hypoxia group cells were seeded in a 24-well plate. When cell confluence reached 90%, the cells were incubated in a mixture of 50 μl of serum-free Opti-MEM-diluted miRNA mimics (2.5 pg) or miRNA inhibitors (2.5 pg) and 50 μl of serum-free Opti-MEM-diluted Lipofectamine 2000 (1 μl; Invitrogen, United States) for 6–8 h in an atmosphere of 5% CO2 at 37°C. When the cells were incubated for 72 h, total RNA was extracted; 72 h later, total protein was extracted. The expression levels of miR-208b, miR-21, TGF-β1 and Smad-3 were detected.

### Reverse Transcription-Quantitative Polymerase Chain Reaction

Total RNA was isolated from transfected cells and plasma by using TRIzol reagent (ABI-Invitrogen, United States). An AUV Spectrophotometer (Nanodrop Lite, Thermo, Germany) was used to measure the A260/A280 value and RNA concentration. RNA was reverse transcribed into cDNA using the SuperScript III cDNA Synthesis Kit (ABI-Invitrogen, United States). qRT-PCR was performed with SYBR qPCR mix (ABI-Invitrogen, United States) using SYBR Green Real-time PCR Applied Biosystems (StepOne Software, United States). The reaction volume was 20 μl, and the reaction conditions were 40 cycles at 95°C for 5 min, 95°C for 10 sec and 58°C for 20 sec. MiRNA expression was measured quantitatively using U6 as an internal control, and the mRNA expression levels of TGF-β1 and Smad-3 were measured using glyceraldehyde 3-phosphate dehydrogenase (GAPDH) as an internal reference gene. The primers used are shown in Table 1. Relative expression was calculated using the 2-ΔΔCt method.

**TABLE 1:** Primers used for RT-qPCR.

Gene	Primer sequences
miR-208b (human)	RT: 5′-GTCGTATCCAGTGCAGGGTCCGAGGTATTC GCACTGGATACGACCTGCCC-3′ F: 5′-AAAAGGTTTGTCTGAGGGCAG-3′ R: 5′- CAGTGCAGGGTCCGAGGTA-3′
miR-208b (cell)	RT: 5′-GTCGTATCCAGTGCAGGGTCCGAGGTATTCG CACTGGATACGACCTGCCC-3′ F: 5′-AAGCTTTTTGCTCGCGTTAT-3′; R: 5′- CAGTGCAGGGTCCGAGGTA-3′
miR-21 (human)	RT: 5′- GTCGTATCCAGTGCAGGGTCCGAGGTATTCG CACTGGATACGACCTCCGT-3′ F: 5′-GTTGTCACTTCCCACAGCACGGAG-3′ R: 5′- CAGTGCAGGGTCCGAGGTA-3′
miR-21 (cell)	RT: 5′- GTCGTATCCAGTGCAGGGTCCGAGGTATTCG CACTGGATACGACCTCCGT-3′ F: 5′-GCCCGCTAGCTTATCAGACTGATG-3′ R: 5′- GTGCAGGGTCCGAGGTGAGGT-3′
TGF-β1 (human):	F: 5′- GGCCAGATCCTGTCCAAGC -3′ R: 5′-GTGGGTTTCCACCATTAGCAC-3′
TGF-β1 (cell):	F: 5′-CTCCCGTGGCTTCTAGTGC-3′ R: 5′- GCCTTAGTTTGGACAGGATCTG-3′
Smad-3 (human):	F: 5′- TGGACGCAGGTTCTCCAAAC-3′ R: 5′- CCGGCTCGCAGTAGGTAAC-3′,
Smad-3 (cell):	F: 5′- ATTCCCGAGAACACTAACTTCCC-3′ R: 5′- TTCATCTGGTGGTCACTGGTT-3′
U6	F: 5′- CGATACAGAGAAGATTAGCATGG -3′ R: 5′- ATATGGAACGCTTCACGAA -3′
GAPDH	F: 5′- TCCTCCTGAGCGCAAGTACTCC-3′ R: 5′- CATACTCCTGCTTGCTGATCCAC-3′

*GAPDH, glyceraldehyde-3-phosphate dehydrogenase; RT-qPCR, reverse transcription-quantitative polymerase chain reaction; TGF-β1, transforming growth factor-β1; F, forward; R, reverse.*

### Western Blot

The cells were washed with phosphate-buffered saline (PBS), followed by the addition of 100 μl of cell lysates and incubation at 4°C for 30 min. Centrifugation was performed at 12,000 x g for 15 min. The supernatant was collected, and the protein concentration was measured using a BCA Kit (MDL, China). The total protein extracted was electrophoresed on a 10% sodium dodecyl sulfate (SDS)-polyacrylamide gel (Bio-Rad, United States). After being transferred onto polyvinylidene fluoride (PVDF) membranes (Millipore, United States), the total protein was sealed with 5% dried skim milk at room temperature for 1 h. After blocking, the samples were treated with primary antibodies against TGF-β1 (1:1000, ab92486) (Abcam, United States), Smad-3 (1:1000, bs-3484R) (Bioss, China), and β-actin (1:500, 4970S) (CST, United States) at 4°C for 12 h. After the membrane was rinsed with TBST (Solarbio, China), the secondary HRP-conjugated antibody was added and incubated in the dark at room temperature for 1 h. Next, after TBST washing, the bands were visualized using the ECL kit (Solarbio, China). The relative expression levels of proteins were obtained via ImageJ 1.53f (NIH, United States). The integral optical density of each strip was calculated, and the ratio of the integral optical density value of the target band with the integrated optical density value of the reference β-actin was the relative expression value of the target protein. This experiment was repeated 3 times.

### Statistical Analysis

The cell experiments were performed in triplicate. Data are expressed as the mean ± SD. GraphPad Prism 8.3.0 (GraphPad LLC, United States) was used for analyses and plotting. The differences were assessed using Student’s *t*-test or one-way analysis of variance (ANOVA). *P* < 0.05 was defined as statistically significant.

## Results

### The Changes in Serum Indices

**FIGURE 1:**
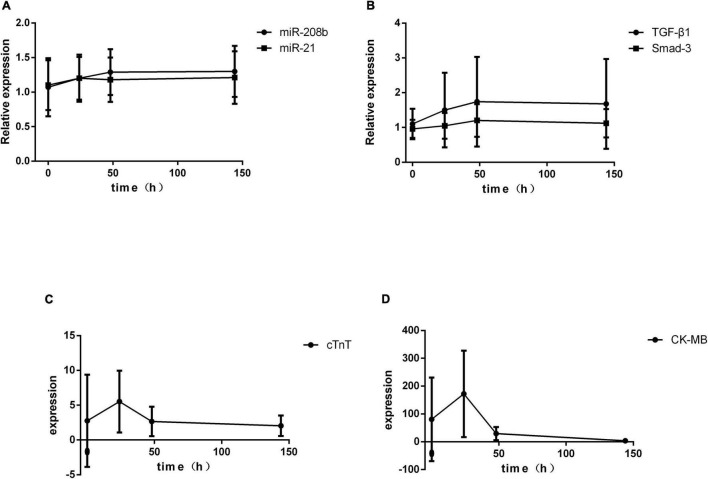
Temporal changes in serum indices in AMI patients. **(A)** The expression levels of miR-208b and miR-21 change with time; **(B)** the expression levels of TGF-β1 and Smad-3 change with time; **(C)** the expression level of cTnT changes with time; **(D)** the expression level of CK-MB changes with time. CK-MB, creatine kinase isoenzyme; cTnT, cardiac calcium protein T; TGF-β1, transforming growth factor-β1.

A total of 36 patients were enrolled. The serum indices of AMI patients were assessed after admission and at 24 h, 48 h and 6 d after onset of the symptoms by qRT-PCR. The results showed significant differences in cTnT and CK-MB expression at different times (*P* < 0.01), and the miR-208b, miR-21, TGF-β1 and Smad-3 levels were not significantly different (Table 2). The trends of change in these indices indicated that cTnT and CK-MB expression peaked at 24 h after onset of the symptoms and then decreased gradually with time, and miR-208b, miR-21, TGF-β1 and Smad-3 expression showed no peak and increased gradually with time (Figure 1).

**TABLE 2:** Expression of serum indices in AMI patients at different time periods.

	Admission	24 h	48 h	6 d	*P*
cTnT	2.76 ± 6.63	5.52 ± 4.44	2.66 ± 2.12	2.04 ± 1.47	<0.01
CK-MB	80.56 ± 149.87	172.34 ± 155.36	29.23 ± 23.48	3.49 ± 2.17	<0.01
miR-208b	1.07 ± 0.42	1.20 ± 0.34	1.29 ± 0.33	1.30 ± 0.37	0.12
miR-21	1.10 ± 0.36	1.20 ± 0.31	1.18 ± 0.32	1.21 ± 0.38	0.68
TGF-β1	1.10 ± 0.44	1.50 ± 1.07	1.74 ± 1.29	1.68 ± 1.29	0.22
Smad-3	0.96 ± 0.26	1.05 ± 0.37	1.20 ± 0.47	1.12 ± 0.41	0.08

*CK-MB, creatine kinase isoenzyme; cTnT, cardiac calcium protein T; TGF-β1, transforming growth factor-β1.*

### The Correlation Between miR-208b/miR-21 and TGF-β1/Smad-3 *in vitro*

**FIGURE 2:**
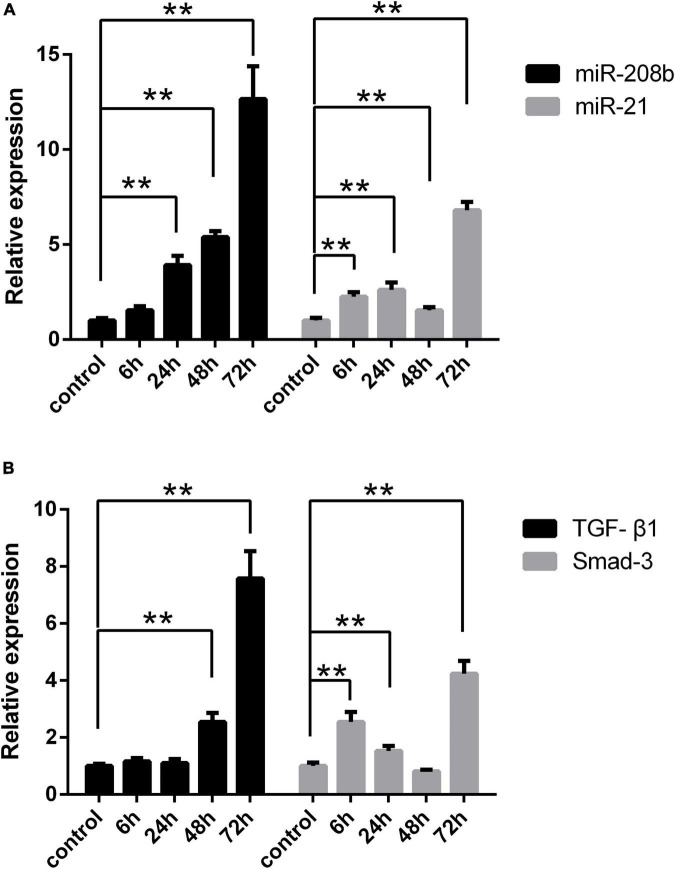
Comparison of the expression of each index under different hypoxia conditions *in vitro*. **(A)** Expression of miR-208b and miR-21 in the hypoxic treatment group at 6 h, 24 h, 48 h, and 72 h detected by RT-qPCR; **(B)** expression of TGF-β1 and Smad-3 in the hypoxic treatment group at 6 h, 24 h, 48 h, and 72 h detected by RT-qPCR. RT-qPCR, reverse transcription-quantitative polymerase chain reaction; TGF-β1, transforming growth factor-β1; *P < 0.05 compared with the control; **P < 0.01 compared with the control.

**FIGURE 3:**
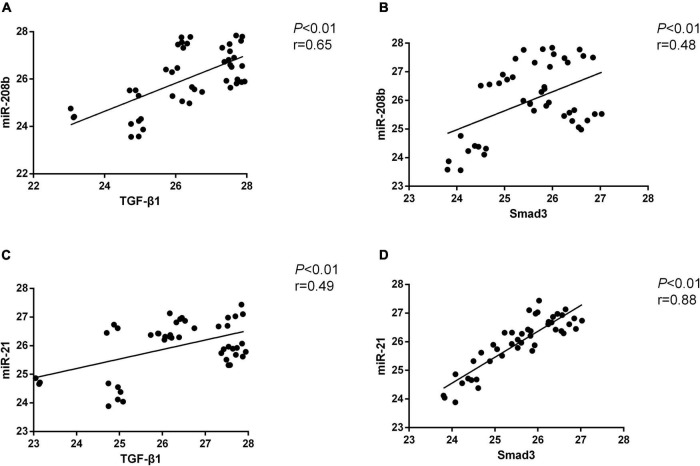
The correlation between miR-208b/miR-21 and TGF-β1/Smad-3. **(A)** Positive correlation between miR-208b and TGF-β1; **(B)** positive correlation between miR-208b and Smad-3; **(C)** positive correlation between miR-21 and TGF-β1; **(D)** positive correlation between miR-21 and Smad-3. TGF-β1, transforming growth factor-β1.

Myocardial H9C2 cells under normal conditions were used as controls, and the same cells were subjected to hypoxia treatment for 6 h, 24 h, 48 h, and 72 h. In general, miR-208b levels were elevated as the time of the hypoxia treatment increased, miR-21 expression peaked at 24 h and 72 h and showed the highest level at 72 h, TGF-β1 levels were also elevated as the time of the hypoxia treatment increased, and Smad-3 expression peaked at 6 h and 72 h and showed the highest level at 72 h (Figure 2). The correlation between miR-208b/miR-21 and TGF-β1/Smad-3 was further analyzed. The results showed a significant correlation between miR-208b and TGF-β1/Smad-3 (*r* = 0.65 and 0.48, *P* < 0.01) and between miR-21 and TGF-β1/Smad-3 (*r* = 0.49 and 0.88, *P* < 0.01) (Figure 3).

### MiR-208b Regulates TGF-β1/Smad-3 Expression

**FIGURE 4:**
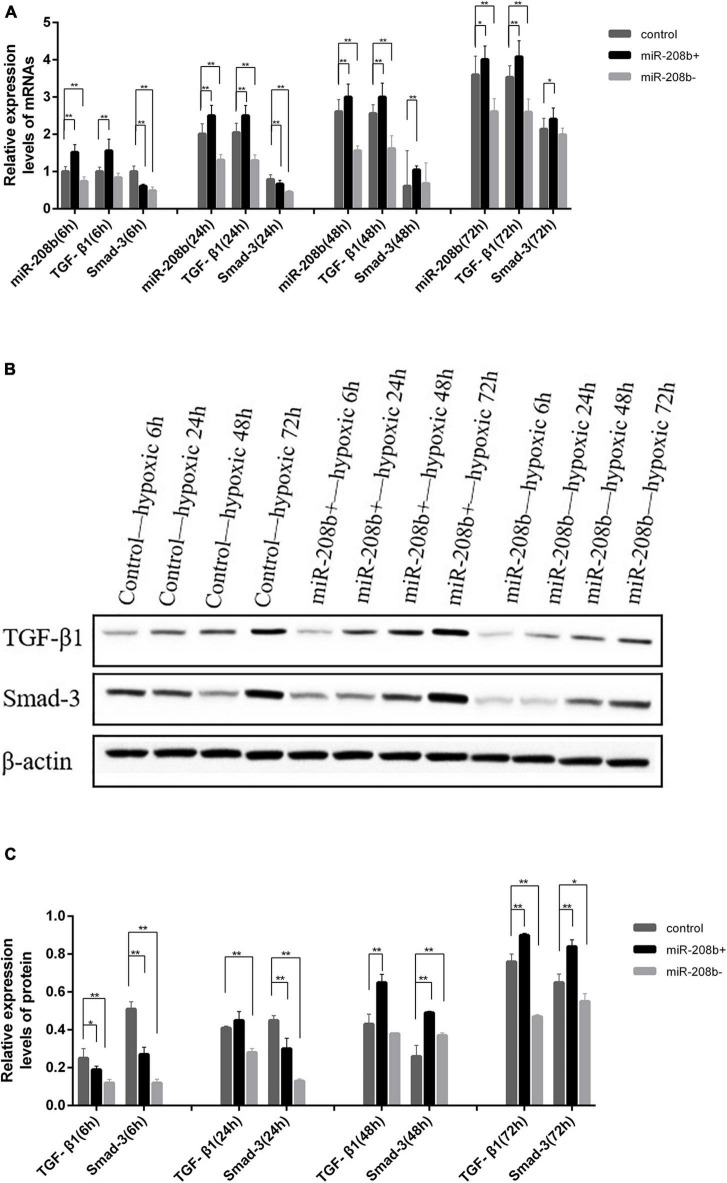
Comparison of the expression of TGF-β1 and Smad-3 in H9C2 cells following transfection with miR-208b mimics and miR-208b inhibitors. **(A)** Comparison of the expression of TGF-β1, Smad-3, and miR-208b detected by RT-qPCR in each group; **(B)** Representative blot images; **(C)** Comparison of the expression of TGF-β1 and Smad-3 detected by western blot analysis in each group. miR-208b +, miR-208b overexpression group; miR- 208b-, miR-208b inhibition group; TGF-β1, transforming growth factor-β1; *P < 0.05 compared with the blank group; **P < 0.01 compared with the blank group.

The TGF-β1/Smad-3 mRNA levels in the miR-208b overexpression, inhibition and control groups were assessed at various times under hypoxic conditions by qRT-PCR. Compared with that of the control group at various times under hypoxic conditions, the expression level of miR-208b was significantly elevated in the miR-208b overexpression group (*P* < 0.01) and significantly reduced in the miR-208b inhibition group (*P* < 0.01). The expression level of TGF-β1 was significantly elevated in the miR-208b overexpression group (*P* < 0.01), reduced in the miR-208b inhibition group, and significantly reduced at 24 h, 48 h, and 72 h (*P* < 0.01). However, the expression level of Smad-3 was significantly reduced in the miR-208b overexpression group at 6 h and 24 h (*P* < 0.01), elevated in the miR-208b overexpression group at 48 h and 72 h (*P* < 0.01, *P* < 0.05), reduced in the miR-208b inhibition group, and significantly reduced at 6 h and 24 h (*P* < 0.01) (Figure 4A). The same trend occurred in western blot analysis; the expression level of TGF-β1/Smad-3 was elevated in the miR-208b overexpression group at 48 h and 72 h and reduced in the miR-208b inhibition group at 6 h, 24 h and 72 h, and this regulation was most obvious at 72 h (Figures 4B,C).

### MiR-21 Regulates TGF-β1/Smad-3 Expression

**FIGURE 5:**
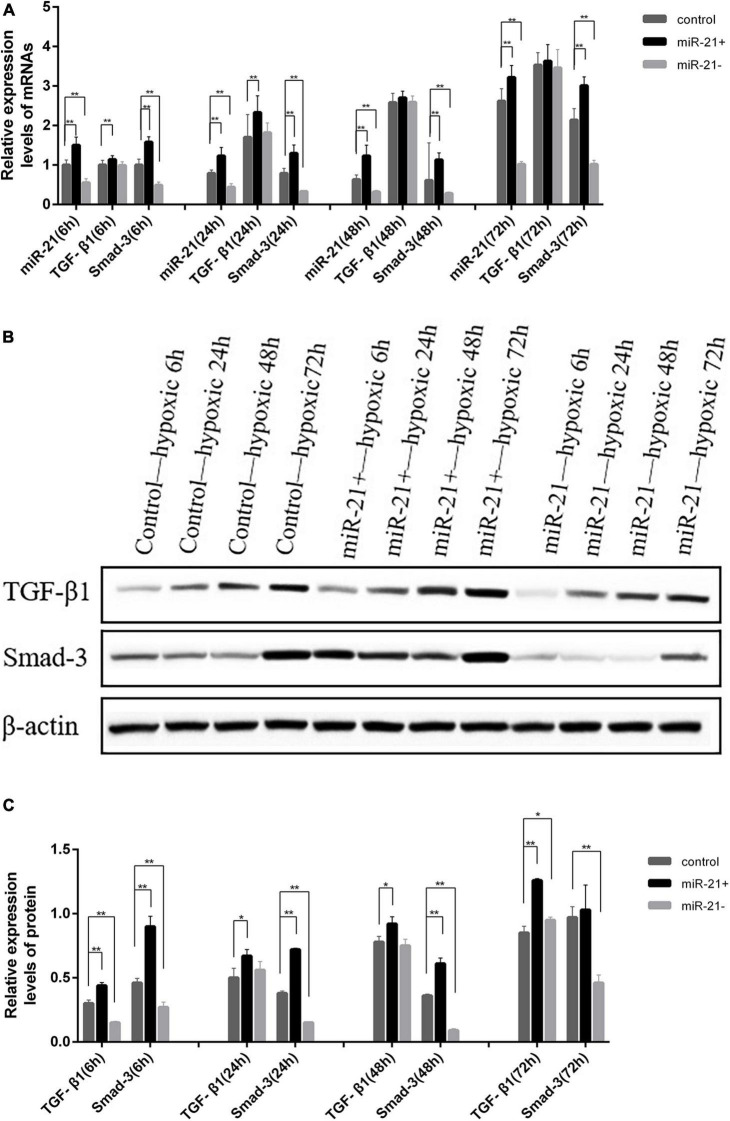
Comparison of the expression of TGF-β1 and Smad-3 in H9C2 cells following transfection with miR-21 mimics and miR-21 inhibitors. **(A)** Comparison of the expression of TGF-β1, Smad-3, and miR-21 detected by RT-qPCR in each group; **(B)** Representative blot images; **(C)** Comparison of the expression of TGF-β1 and Smad-3 detected by western blot analysis in each group. miR-21 +, miR-21 overexpression group; miR- 21-, miR-21 inhibition group; TGF-β1, transforming growth factor-β1; *P < 0.05 compared with the blank group; **P < 0.01 compared with the blank group.

The TGF-β1/Smad-3 mRNA levels in the miR-21 overexpression, inhibition and control groups were determined at various times under hypoxic conditions by qRT-PCR. Compared with that of the control group at various times under hypoxic conditions, the expression level of miR-21 was significantly elevated in the miR-21 overexpression group (*P* < 0.01) and significantly reduced in the miR-21 inhibition group (*P* < 0.01). The expression level of TGF-β1 was elevated in the miR-21 overexpression group, significantly elevated at 6 h and 24 h (*P* < 0.01), and reduced in the miR-21 inhibition group. The expression level of Smad-3 was significantly elevated in the miR-21 overexpression group (*P* < 0.01) and significantly reduced in the miR-21 inhibition group (*P* < 0.01) (Figure 5A). The same trend occurred in western blot analysis; the expression level of TGF-β1/Smad-3 was elevated in the miR-21 overexpression group and reduced in the miR-21 inhibition group (Figures 5B,C).

## Discussion

Cardiac fibrosis can be divided into two categories, replacement fibrosis and reactive fibrosis, and both of these processes are regulated by myofibroblasts. Replacement fibrosis (scar) plays an important role in preventing ventricular wall rupture after AMI (11), but increased intraventricular pressure and paracrine and endocrine factors will promote the formation of connective tissue in non-infarct zones, which will lead to reactive fibrosis. This process will alter myocardial compliance, increase ventricular wall stiffness and then affect cardiac contractility and relaxation, which will ultimately induce arrhythmia (12) and lead to sudden cardiac death (13). As a prelude to scarring, neutrophils are gradually replaced by macrophages (M2 phenotype), lymphocytes, natural killer cells, dendritic cells and more important myofibroblasts. Moreover, the secretion of the anti-inflammatory factors IL-10 and TGF-β in infarct zones gradually increases (14–16), and TGF-β is a central mediator involved in the fibrotic phase of infarcted healing and exerts a biochemical effect by activating Smad-3 (17).

MiRNAs play an important role in heart development and the pathogenesis of heart disease. Among them, miR-21 has been studied most in recent years and is suggested as a predictive and diagnostic maker for many cardiac disorders, including heart failure, myocardial infarction, and coronary artery disease (18–21). Recent studies have shown that miR-21 is involved in cardiac fibrosis. Studies by He et al. suggest that Smad7 is an essential direct target gene of miR-21 and that miR-21 promotes fibrosis by down-regulating Smad7 expression *in vivo* (22). Spry1 is also the target gene of miR-21, and miR-21 and TGF-β1 levels are increased simultaneously in fibroblasts of the failing heart; Spry1 is downregulated when TGF-β1 is overexpressed, thereby controlling the degree of interstitial fibrosis (23). However, it is unclear whether miR-21 is involved in myocardial fibrosis through other pathways.

The miR-208 family consists of miR-208a, miR-208b, and miR-499 (24). As a specific biomarker of myocardial injury (25), miR-208 is involved in cardiac fibrosis, myocardial hypertrophy, heart failure and myocardial ischemia by regulating the conversion of the α-MHC and β-MHC subunits (5, 26). Studies have shown that miR-208a aggravates the ischemic injury of cardiomyocytes by targeting ON CHD9 and NLK (27, 28). Tony et al. also showed that miR-208a can promote the apoptosis of ischemic cardiomyocytes and that silencing miR-208a can suppress the apoptosis, fibrosis and hypertrophy of cells after myocardial infarction (29). Although there are studies suggest that miR-208b protects H9c2 cells from hypoxia-induced apoptosis via targeting Bax and activating PI3K/AKT pathway (30), the specific molecular mechanism of miR-208b’s involvement in myocardial fibrosis remains unclear. Our previous study examined miR-208b expression levels in plasma from 140 AMI patients, and the results suggested that miR-208b could be used as a possible diagnostic marker for the early stages of AMI and an independent predictor of 3-year major adverse cardiovascular events, which was consistent with previous studies suggesting that miR-208b might be a potential biomarker for MI diagnosis and prognosis evaluation.

The aim of this study was to analyze the expression level changes of miR-208b and miR-21 in AMI patients and the regulatory effect on cardiac fibrosis via the TGF-β1/Smad-3 signaling pathway. According to the results, there was no peak in miR-208b, miR-21, TGF-β1 or Smad-3 expression in AMI patients at different times but a trend of increase over time. Subsequent *in vitro* cell experiments suggested that miR-208b and TGF-β1 expression increased gradually with prolonged hypoxia time, which is consistent with the results of the plasma analysis. MiR-21 and Smad-3 expression peaked at 24 h and 72 h and at 6 h and 72 h, respectively, and the expression at 72 h was highest, which is also consistent with the results of the plasma analysis. This result was mainly because the longer the duration of the hypoxic-ischemia, the more severe the myocardial tissue and cell injury was, and the higher the levels of miR-208b, miR-21, TGF-β1 and Smad-3 were.

Studies have confirmed that TGF-β is a target gene of miR-21, and TGF-β-induced endothelial cell transition to mesenchymal cells has been shown to partly occur through miR-21-mediated signaling pathways (31, 32). Cell and animal studies by Ma et al. showed that miRNA-21 promotes the progression of peritoneal fibrosis through the activation of the TGF-β/Smad signaling pathway (33). Another intervention study in cardiac fibroblasts by Yu et al. showed that miRNA-33a deficiency inhibits proliferation and fibrosis through inactivation of the TGF-β/Smad pathway (34). In this study, miRNA mimics and inhibitors were used to transfect H9C2 myocardial cells to construct miRNA inhibition or overexpression groups, cells under different hypoxic culture times were used to simulate ischemic conditions, and qRT-PCR was used to assess the expression at the gene level. Our results indicated that the expression levels of TGF-β1 and Smad-3 increased in the miR-208b and miR-21 overexpression groups and decreased in the miR-208b and miR-21 inhibition groups. In addition, the longer the hypoxic time was, the greater the difference was. Western blot results demonstrated the same trend at the protein level. Therefore, under anoxic conditions, the expression level of miR-208b/miR-21 increased, and miR-208b/miR-21 promoted the progression of cardiac fibrosis through the activation of the TGF-β1/Smad-3 signaling pathway.

## Conclusion

In conclusion, this study demonstrated that miR-208b/miR-21 may be involved in the progression of cardiac fibrosis by upregulating the expression of TGF-β1/Smad-3. Thus, targeting miR-208b/miR-21 may be an effective therapy for cardiac fibrosis. However, this study has certain limitations. We analyzed at the cellular and molecular levels, and animal studies are needed.

## Data Availability Statement

The original contributions presented in the study are included in the article/supplementary material, further inquiries can be directed to the corresponding author.

## Ethics Statement

The studies involving human participants were reviewed and approved by Ethics Committee of Xi’an No. 9 Hospital. The patients/participants provided their written informed consent to participate in this study. Written informed consent was obtained from the individual(s) for the publication of any potentially identifiable images or data included in this article.

## Author Contributions

ZF conceived and designed the experiments. YZ and BY performed the experiments. NZ analyzed and interpreted the data. RZ and LL contributed reagents, materials, analysis tools or data. YZ and YX wrote the manuscript.

## Conflict of Interest

The authors declare that the research was conducted in the absence of any commercial or financial relationships that could be construed as a potential conflict of interest.

## Publisher’s Note

All claims expressed in this article are solely those of the authors and do not necessarily represent those of their affiliated organizations, or those of the publisher, the editors and the reviewers. Any product that may be evaluated in this article, or claim that may be made by its manufacturer, is not guaranteed or endorsed by the publisher.
